# Preclinical evidence of the enhanced effectiveness of combined rapamycin and AICAR in reducing kidney cancer

**DOI:** 10.1002/1878-0261.12370

**Published:** 2018-10-12

**Authors:** Sitai Liang, Edward A. Medina, Boajie Li, Samy L. Habib

**Affiliations:** ^1^ Department of Cell Systems & Anatomy University of Texas Health Science Center at San Antonio TX USA; ^2^ Department of Pathology and Laboratory Medicine University of Texas Health Science Center at San Antonio TX USA; ^3^ Bio‐X Institutes Shanghai Jiao Tong University China; ^4^ South Texas Veterans Health Care System San Antonio TX USA

**Keywords:** 5‐aminoimidazole‐4‐carboxamide‐riboside (AICAR), hypoxia‐inducible factor (HIF‐α), kidney cancer, rapamycin, Von Hippel‐Lindau

## Abstract

Loss of Von Hippel‐Lindau in renal carcinoma cells results in upregulation of the activity of hypoxia‐inducible factor (HIF‐α), a major transcription factor involved in kidney cancer. Rapamycin as mammalian target of rapamycin inhibitor and 5‐aminoimidazole‐4‐carboxamide‐riboside (AICAR) as AMPK activator are used separately to treat cancer patients. In the current study, the possible additive effect of drug combinations in reducing kidney tumorigenesis was investigated. Treatment with drug combinations significantly decreased cell proliferation, increased cell apoptosis, and abolished Akt phosphorylation and HIF‐2α expression in renal cell carcinoma cells, including primary cells isolated from kidney cancer patients. Significant decreases in cell migration and invasion were detected using drug combinations. Drug combinations effectively abolished binding of HIF‐2α to the Akt promoter and effected formation of the DNA‐protein complex in nuclear extracts from 786‐O cells, as demonstrated using electromobility shift assay and examination of Akt promoter activity. Importantly, we tested the effect of each drug and the combined drugs on kidney tumor size in the nude mouse model. Our data show that treatment with rapamycin, AICAR, and rapamycin+AICAR decreased tumor size by 38%, 36%, and 80%, respectively, suggesting that drug combinations have an additive effect in reducing tumor size compared with use of each drug alone. Drug combinations effectively decreased cell proliferation, increased apoptotic cells, and significantly decreased p‐Akt, HIF‐2α, and vascular endothelial growth factor expression in tumor kidney tissues from mice. These results show for the first time that drug combinations are more effective than single drugs in reducing kidney tumor progression. This study provides important evidence that may lead to the initiation of pre‐clinical trials in patients with kidney cancer.

**Abbreviations**
AICAR: 5‐aminoimidazole‐4‐carboxamide‐riboside
AMPK: 5′ AMP‐activated protein kinase
BW: bodyweight
CMV: cytomegalovirus
DMEM: Dulbecco's modified Eagle's medium
EMSA: electrophoretic mobility shift assay
GSH: glutathione
GST: glutathione‐*S*‐transferase
HIF‐α: hypoxia‐inducible factor
i.p.: intraperitoneal
mTOR: mammalian target of rapamycin
PCNA: proliferating cell nuclear antigen
PCNA: proliferating cell nuclear antigen
PI: propidium iodide
RCC: renal cell carcinoma
TCA: trichloroacetic acid
VEGF: vascular endothelial growth factor
VHL: Von Hippel‐Lindau

## 1 Introduction

Renal cell carcinoma (RCC) is one of the most lethal kidney cancers in the USA (Kabaria *et al*., 2016). RCC represents a class of renal epithelial tumors with distinct histological subtypes, including clear cell, papillary and chromophobe renal carcinoma, and renal oncocytomas; histological classification has critical implications for prognosis and treatment (Muglia and Prando, 2015). Approximately 50% of patients with metastatic RCC have a survival rate of < 1 year (Muglia and Prando, 2015). Although most RCCs occur sporadically, several genes have been linked to an inherited predisposition to kidney cancer; these genes may also play a role in sporadic renal cancer (Muglia and Prando, 2015; Schmidt and Linehan, 2016).

Loss or inactivation of the tumor suppressor gene Von Hippel‐Lindau (*VHL*) in renal cancer cells results in high levels of hypoxia‐inducible factors (HIF) activity, a major transcription factor involved in kidney tumorigenesis (Riazalhosseini and Lathrop, 2016). Increased basal HIF‐1α and HIF‐2α can stimulate angiogenesis and promote the development of RCCs and hemangioblastomas (Cuvillier, 2017). Although HIF‐1α and HIF‐2α seem to be regulated similarly, evidence suggests that the two α subunits exhibit distinct roles in hypoxic expression and activation of target genes (Franovic *et al*., 2009; Majmundar *et al*., 2010). HIF protein expression is regulated by various stimuli, including changes in cellular oxygen concentration, growth factors, and oncogenic activation or loss of tumor suppressor gene function (Carroll and Ashcroft, 2006; Samanta *et al*., 2014). Overexpression of HIF‐1α and HIF‐2α leads to the upregulation of genes involved in proliferation, angiogenesis, and glucose metabolism, and is associated with tumor progression in several cancers including RCC (Gordan *et al*., 2008; Rankin and Giaccia, 2008). Under normal oxygen tension, the α subunits are hydroxylated at conserved prolyl and asparaginyl residues and are targeted for degradation by the VHL ubiquitin E3 ligase complex (Safran and Kaelin, 2003).

Akt, or protein kinase B (PKB), is a major survival protein kinase involved in several types of cancers (Ackah *et al*., 2005; Altomare and Testa, 2005; Bago *et al*., 2016; Brunet *et al*., 1999; Calamito *et al*., 2010). Akt phosphorylated by propidium iodide (PI)‐3K at Ser^473^ phosphorylates/inactivates *TSC2*, which leads to the activation of the mammalian target of rapamycin (mTOR; Sarbassov *et al*., 2005). mTOR is constitutively activated in many types of human cancers (Dowling *et al*., 2010; Inoki *et al*., 2005; Thoreen *et al*., 2009; Wagle *et al*., 2014a,b). Activation of the Akt/mTOR signaling pathway positively regulates HIF (Woo *et al*., 2015), making mTOR inhibitors attractive anticancer agents. The use of mTOR inhibitors in the treatment of various types of cancer has been actively studied both preclinically and clinically (Jacinto *et al*., 2004; Mayer and Grummt, 2006; Pan *et al*., 2017; Sarbassov *et al*., 2006). On the other hand, AMP kinase (AMPK) is the primary energy sensor in cells that activates tumor suppressor genes to block HIF activity (Tong *et al*., 2011). The AMPK activator 5‐aminoimidazole‐4‐carboxamide‐riboside (AICAR) inhibits the growth and survival of cancer cells (Guo *et al*., 2009). We recently reported that inhibiting mTOR with rapamycin or activating AMPK with AICAR upregulates the DNA repair pathway in kidney cancer cells and in the *TSC2* ^*+/−*^ mouse model. These data suggest one mechanism whereby rapamycin might inhibit the formation and progression of kidney cancer through activation of DNA repair pathway (Habib *et al*., 2010a,b).

Rapamycin and AICAR have been approved for the treatment of several diseases including cancer, and each is currently being used in clinical studies. Here, we investigated the possibility of combining rapamycin and AICAR to inhibit additively the Akt/HIF‐2/VEGF pathway and reduce kidney tumor progression in athymic nude mice injected with 786‐O cells (under the kidney capsule) compared with treatment with each drug alone.

## 2 Materials and methods

### 2.1 Cell culture

#### 2.1.1 Renal cancer cells

Human *VHL*‐deficient (786‐O) and ACHN cells were grown in Dulbecco's modified Eagle's medium (DMEM) containing 10% FBS, 5.5 mm glucose, 100 units·mL^−1^ of penicillin, 100 μg·mL^−1^ of streptomycin, and 2 mmol·L^−1^ of glutamine. Cells confluences at about 80% were growth‐arrested overnight in serum‐free DMEM before experiments.

#### 2.1.2 Cell treatment

HRCC, 786‐O, and ACHN cells were grown to 80–90% confluence in 60‐mm Petri dishes. Cells were treated with serial concentrations of AICAR (0, 2, 4, 10 mm), rapamycin (0, 20, 40, 100 nm) or drug combinations (0/0, 2/20, 4/40, 10/100, mm/nm, AICAR/rapamycin) for 72 h. Cells were treated with drug combinations (2 mm/20 nm, AICAR/rapamycin) for 24, 48, and 72 h. AICAR was obtained from Cayman Chemical (Ann Arbor, MI, USA) and rapamycin from Sigma‐Aldrich (St. Louis, MO, USA). The cells were lysed in lysis buffer and used for Western blot analysis (Simone *et al*., 2008).

#### 2.1.3 Thymidine assay

^3^H‐thymidine incorporation assay was performed on 786‐O and ACHN cells treated with serial concentrations of rapamycin, AICAR or drug combinations as described above for 72 h, or drug combinations (2 mm/20 nm, AICAR/rapamycin) for 24, 48, and 72 h. Cells were washed twice with PBS and then fixed with trichloroacetic acid (TCA) for 30 min at room temperature. TCA was aspirated and the precipitate was lysed in 1 m NaOH for another 30 min and neutralized with 1 m HCl. The cell lysates were transferred into vials contained 5 mL liquid scintillation counting solution and ^3^H‐thymidine incorporation was counted using a beta scintillation counter.

#### 2.1.4 Flow cytometric analysis of apoptosis

The apoptotic cells were measured using annexin V‐FITC conjugated to PI by flow cytometry. Serum‐starved 786‐O and ACHN cells were treated with serial concentrations of rapamycin, AICAR or drug combinations for 72 h. Harvested cells were assayed for apoptosis using an annexin V‐FITC apoptosis detection kit (Calbiochem, Burlington, MA, USA) as previously described (Velagapudi *et al*., 2011). To prepare samples for flow cytometry, cells were gently washed two times with annexin‐binding buffer. To each plate, 0.5 mL of trypsin (0.05%) was added, and the plates were incubated until the cells appeared detached by microscopic evaluation. Cells were released from the plate by gentle tapping and added to the collected cells from the medium. Cells were suspended in cold binding buffer and stained with annexin V‐FITC and PI. Analysis was conducted for 20 000 cells using a flow cytometer with cell quest software (Becton‐Dickinson, Rutherford, NJ, USA).

### 2.2 Western blot analysis

Homogenates of kidney tumor tissues or cell lysates were prepared as previously described (Abuaboud *et al*., 2017). Protein concentrations were determined with the Bradford assay (Bradford, 1976) using BSA as a standard. Western blot analysis was performed as previously described (Habib *et al*., 2010a,b). Phospho‐Akt at Ser^473^, Akt, proliferating cell nuclear antigen (PCNA), cyclin D1, HIF 1/2‐α, and caspase 3 antibodies were from Cell Signaling Technology (Danvers, MA, USA). PARP, VEGF, actin, and GAPDH antibodies were obtained from Santa Cruz Biotechnology (Santa Cruz, CA, USA). An enhanced chemiluminescence kit (Amersham, Piscataway, NJ, USA) was used to identify protein expression. Expression of each protein was quantified by densitometry using National Institutes of Health image 1.62 software and normalized to a loading control.

### 2.3 Construction of Akt promoter‐luciferase reporter plasmid containing HIF‐2α binding sites

We have cloned a section of the *Akt* promoter region (−1 to −1991 relative to translational start site) that contains a potential binding HIF‐2α site into the luciferase reporter vector (pGL3). Forward primers were used as: 5′‐GGTGCCCGAAGCTTCCGCGACGCT‐3′ and reverse primers as: 5′‐GGCCACAGAGCTCCTCAGCAGTCCCAG‐3′. Akt promoter reporter plasmid was used to determine the transcriptional activity of the HIF‐2α gene (Dihlmann *et al*., 2005). A *Renilla* reporter plasmid was used as transfection control. Plasmids were transfected into 786‐O or HRCC cells using the LipofectAMINE and Plus Reagent method (Life Technologies, NY, USA). LipofectAMINE was added to the complex of DNA and Plus reagent and incubated for 15 min at room temperature. DNA and Plus reagent–LipofectAMINE complexes were added to each well and incubated at 37 °C with 5% CO_2_. After incubation for 3–4 h, 1 mL of fresh media with 20% serum was added to a final concentration of 10%. Cells were pretreated with rapamycin (20 nm), AICAR (20 mm) or drug combinations for 72 h. At 48 h after transfection, cells were harvested for Firefly and Renilla luciferase assay using the Dual‐Luciferase Reporter assay kit (Promega, Madison, WI, USA). Luciferase activity was determined using the Luciferase Reporter Assay System by a luminometer according to the manufacturer's instructions (Promega) and normalized by Renilla activity.

### 2.4 Electrophoretic mobility shift assays (EMSA)

Nuclear proteins were extracted from 786‐O cells using nuclear and cytoplasmic extraction kits (Thermo Fisher Scientific, Pierce, IL, USA). The protein concentration of the nuclear extracts was determined using the Bradford method (Bradford, 1976). EMSA binding reactions were performed as previously described (Habib *et al*., 2008a). As control, 20 fmol of the end‐labeled double‐stranded 48‐bp oligonucleotide covering the region of binding of HIF‐2α to Akt promoter from −110 to −61 forward primers (5′‐CCCCCAGGCACGTGCAGTGGGTCT‐3′ and reverse primers (5′‐AGACCCACTGCACGTGCCTGGGGG‐3′) was used. Mutant forward primer (5′‐CCCCCAGGCAAAAACAGTGGGTCT‐3′) and reverse primers (5′‐AGACCCACTGTTTTTGCCTGGGGG‐3′) were incubated with the nuclear extracts isolated from 786‐O cells and 1 μL of poly(dI‐dC)·(dI‐dC). To test the specificity of HIF‐1/2α binding to the Akt promoter, 5 μL of antibody against HIF‐1α and HIF‐2α (Cell Signaling Technology) was pre‐incubated with nuclear extracts. Anti‐IgG antibody (Santa Cruz Biotechnology) was also used as a control antibody. The reactions were carried out at room temperature for 30 min prior to addition of the radiolabeled probe. Competition assays were performed in the presence of a 100‐fold excess of the unlabeled oligonucleotide. The complexes were resolved using a 5% non‐denaturing polyacrylamide gel. The gels were dried and exposed overnight at −70 °C.

### 2.5 Migration and invasion assays

To test the effect of each drug or drug combinations on cell invasion and cell migration, 786‐O cells treated for 72 h with rapamycin (20 nm), AICAR (2 mm) or drug combinations (20 nm rapamycin/2 mm AICAR) were added into the upper well chamber of a 96‐well plate (Millipore, Burlington, MA, USA) and the lower chamber filled with DMEM. Following 72 h of incubation, each well was washed with PBS. The migrated and invaded cells in the lower chamber were fixed with methanol and stained with 0.1% crystal violet solution for 10–15 min. Each well was washed with PBS, and upper well cells were scrapped with wet cotton and lower well cells stained. The total number of migrated and invaded cells was counted using counting software and the images of migrated or invaded cells were taken using a Nikon (Melville, NY, USA) light inverted microscope.

### 2.6 Generation of stable RCC cell line expressing luciferase

786‐O cells were stably transfected with the pGL3‐control vector (Promega) and with pSV2Neo vector (from The American Type Culture Collection, Manassas, VA, USA), as previously described (Edinger *et al*., 1999). Cells were treated with Lipofectamine 2000 and plus reagent (Invitrogen, Carlsbad, CA, USA) in Opti‐MEM and selected with geneticin (400 μg·mL^−1^). Several stable clones expressing Luc were isolated and tested for luciferase activity. Luciferase activity was determined using the Luciferase Reporter Assay System by luminometer. The clones expressing the highest levels of luciferase were selected and tested *in vivo* using a IVIS, PerkinElmer bioluminescence Imaging Systems (Waltham, MA, USA). One million 786‐O cells stably expressing high luciferase activity of Akt promoter were injected into the kidney capsule of 5‐week‐old nude mice. Tumor growth in all groups was evaluated by measuring the emitted luminescence using a bioluminescence imager following injection of luciferin. Treatment with AICAR, rapamycin or drug combinations was started when the average tumor volume reached 50 μm^3^. AICAR, rapamycin or both drugs were injected intraperitoneally (i.p.) (2 mg·kg^−1^ bodyweight (BW) of rapamycin, 250 mg·kg^−1^ BW of AICAR or drug combinations) for 5 days/week for 4 weeks. Tumor size was measured every week during the drug injections using the PerkinElmer bioluminescence imaging systems and compared with tumor size in non‐treated animals. Mice were sacrificed after 4 weeks of drug treatments, and tumor size measured and then dissected from the kidneys of non‐treated and treated mice.

### 2.7 Animals

#### 2.7.1 Nude mice

We have established several clones of 786‐O cells expressing luciferase driven by the cytomegalovirus (CMV) promoter. One million VHL‐deficient (786‐O) cells expressing luciferase were injected under the kidney capsule of 5‐week‐old athymic nude male mice. Animals were purchased from Harlan Laboratories. The study has been approved by the Institutional Review Board of The University of Texas Health Science Center at San Antonio, TX, USA. The animals were allowed food and water *ad libitum* prior to and during the experiments. Mice were divided into four groups of five mice. Group 1 mice (control) were injected with an appropriate amount of vehicle (DMSO). Group 2 mice were injected i.p. with 2 mg·kg^−1^ BW rapamycin in DMSO 5 days/week for 4 weeks as previously described (Habib *et al*., 2010b). Group 3 mice were injected with 250 mg·kg^−1^ BW AICAR in DMSO 5 days/week for 4 weeks as previously described (Theodoropoulou *et al*., 2013). Group 4 mice were injected with the same doses of rapamycin and AICAR in DMSO 5 days/week for 4 weeks. Injections were carried out under isoflurane inhalation anesthesia (Abbott, Abbott Park, IL, USA). Ten minutes prior to imaging, animals were injected with luciferin (Xenogen, Perkin Elmer, OH, USA) (120 mg·kg^−1^, i.p.). Total photon flux/s in the tumor area measured by bioluminescent imaging and bioluminescent signals was recorded weekly using the Xenogen IVIS System. Animals were euthanized and kidneys were removed rapidly to measure tumor size by two independent observers blinded to the experimental conditions. The xenografted tumors were formalin‐fixed, paraffin‐embedded, and tissue blocks were serially sectioned. In addition, part of the kidney tumor was used for biochemical and immunofluorescence staining analysis.

### 2.8 Nephrotoxicity assay

Urinary excretion of glutathione‐*S*‐transferase (GST), an indicator of the loss of cell membrane integrity, was measured in urine collected in metabolic cages for all mice groups. GST was measured using dinitrochlorobenzene and glutathione (GSH) as substrate and co‐substrate, respectively (Walshe *et al*., 2009). One unit of GST activity represents the formation of 1 μmol of dinitrochlorobenzene GSH conjugate/mL/min at pH 6.5 and 25 °C.

### 2.9 Immunoperoxidase staining of Ki67 and VEGF

Detection of Ki67 and VEGF was performed on paraffin kidney tumor sections from mice by immunoperoxidase staining as previously described (Liang *et al*., 2013). Sections were incubated with rabbit anti‐Ki67 or VEGF antibody (Abcam, Cambridge, MA, USA) for 30 min, washed twice with PBS, and incubated with horseradish peroxidase‐labeled anti‐rabbit antibody for 30 min. The horseradish peroxidase was developed with diaminobenzidine tetrahydrochloride and hydrogen peroxide in PBS. To demonstrate staining specificity, control kidney sections were stained without primary antibody. Immunostained sections were viewed and photographed using a Nikon microscope equipped for epifluorescence with excitation and bandpass filters.

### 2.10 TUNEL assay

TUNEL staining using the TUNEL Apoptosis Detection Kit (Upstate, Burlington, MA, USA) was performed as previously described (Velagapudi *et al*., 2011). Sections were examined using light microscopy. Sections incubated with PBS, instead of terminal deoxynucleotidyl transferase (TdT) enzyme solution, served as negative controls. The number of TUNEL‐positive cells was counted in five randomly selected fields under 40× magnification in each kidney tumor section from each animal.

### 2.11 Immunofluorescence staining of HIF‐2α

A fluorescent labeling method of HIF‐2α immunostaining was used on kidney tumor sections from mice as described previously (Habib *et al*., 2008a,b). FITC green signals for HIF‐2α were detected using a filter with an excitation range of 450–490 nm and a filter emission at 535 nm. Kidney sections were viewed and photographed using a Nikon Research microscope equipped for epifluorescence with excitation and bandpass filters. To demonstrate staining specificity, control kidney sections were stained without primary antibody.

### 2.12 Statistics

Data are presented as the mean ± SEM. Statistical differences were determined using ANOVA followed by Student Dunnett's (Exp. vs. Control) test using one‐trial analysis. *P‐*values less than 0.01 were considered statistically significant.

## 3 Results

### 3.1 Low‐dose drug combinations of rapamycin and additive induce cell apoptosis

**Figure 1:**
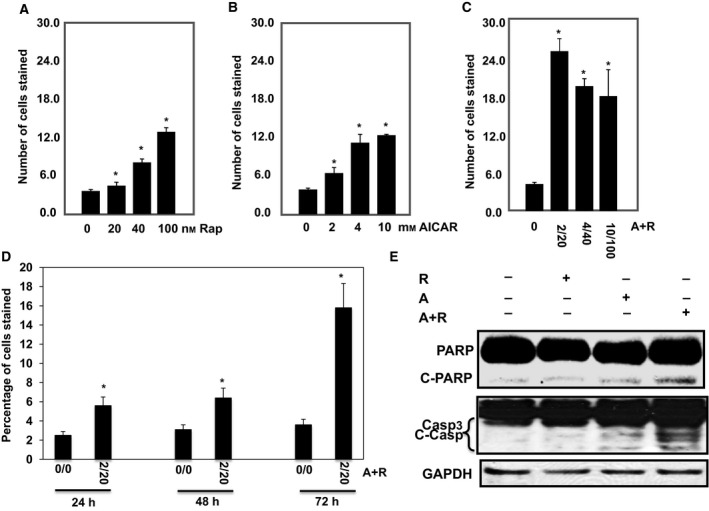
Significant increase in number of apoptotic cells is dependent on drug concentration and time exposure in 786‐O cells. Serial concentrations of (A) rapamycin (0–100 nm), (B) AICAR (0–10 mm), and (C) drug combinations (0/0–2/20, 4/40 and 10/100, mm/nm) show that increase in number of apoptotic cells is dose‐dependent using annexin V‐FITC conjugated to PI by flow cytometry. In addition, treatment of the cells with drug combinations for 24, 48, and 72 h (D) shows that increase in number of apoptotic cells is time‐dependent. Apoptotic data were confirmed in cells by measuring apoptotic protein expression. Lysates from cells treated with rapamycin (20 nm), AICAR (2 mm) or rapamycin+AICAR (20 nm/2 mm) for 72 h were subjected to Western blot analysis to measure PARP and caspase 3 cleavages. (E) Significant increase was detected in cleavage of PARP at 85 kDa and caspase 3 at 22 and 17 kDa in 786‐O cells treated with drug combinations compared with cells treated with each drug alone for 72 h. GAPDH was used as a loading control. Data represent mean ± SEM (*n* = 4). Significant difference from control tissues is indicated by *P <* *0.01.

To test the effective dose of each drug or the additive effect of drug combinations on cell apoptosis, cells were treated with serial concentrations of AICAR (0–10 mm), rapamycin (0–100 nm) or a combination of both drugs (2/20, 4/40 or 10/100, mm/nm) for 72 h. 786‐O and ACHN cells treated with rapamycin or AICAR exhibited a dose‐dependent increase in the number of apoptotic cells with a maximum threefold increase in the number of apoptotic cells at the highest dose of each drug compared with non‐treated cells (Figs 1A,B and S1). On the other hand, the most effective low dose of combined drugs (2/20, mm/nm) showed a sevenfold increase in the number of apoptotic cells compared with non‐treated cells (Figs 1C and S1). In addition, cells treated with this drug combination (2 mm/20 nm, AICAR/rapamycin) for different time periods (24, 48, and 72 h) demonstrated time‐dependent apoptosis (Figs 1D and S1). We also confirmed the induction of apoptosis proteins (PARP and caspase 3 cleavage) for cells treated with each drug and the more pronounced increase for cells treated with the drug combination (Figs 1E and S3).

### 3.2 Drug combinations of rapamycin and AICAR additively decrease cell proliferation

**Figure 2:**
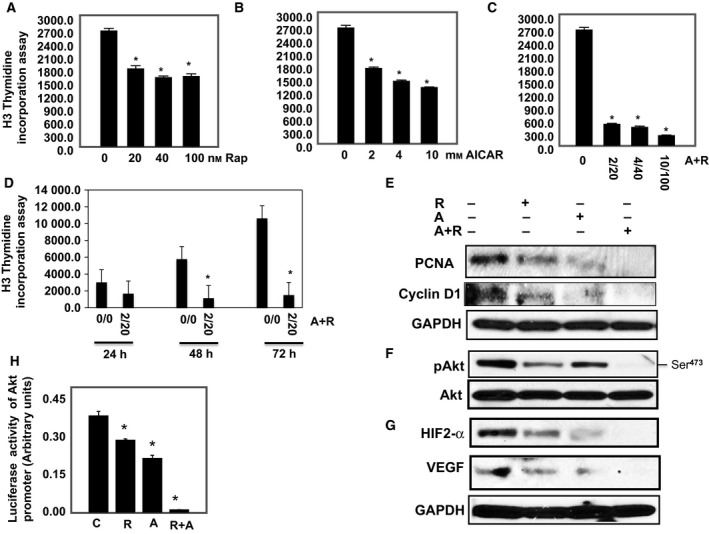
Decrease in cell proliferation by drug combinations is dependent on the drug concentration and duration of exposure in 786‐O cells. Serial concentrations of (A) rapamycin (0–100 nm), (B) AICAR (0–10 mm), and (C) drug combinations (0/0–2/20, 4/40 and 10/100, mm/nm) show that the decrease in cell proliferation is dose‐dependent using the ^3^H‐thymidine incorporation assay. In addition, treatment of the cells with drug combinations for 24, 48, and 72 h (D) show that the significant decrease in cell proliferation is time‐dependent. Cell proliferation data were confirmed in cells by measuring proliferative protein expression. Lysates from cells treated with rapamycin (20 nm), AICAR (2 mm) or rapamycin+AICAR (20 nm/2 mm) for 72 h were subjected to Western blot analysis to measure PCNA and cyclin D1. (E) A significant decrease in expression of PCNA and cyclin D1 was detected in cells treated with a single drug, whereas drug combinations abolished the expression of both proteins detected in cells, providing evidence of the additive effect of drug combinations in reducing cell proliferation. A combination of drugs abolished Akt survival kinase and blocked HIF‐2α and VEGF in 786‐O cells. Cells were treated with rapamycin (20 nm), AICAR (2 mm) or drug combination (20 nm rapamycin/2 mm AICAR) for 72 h. Cell lysates were subjected to Western blot analysis to measure p‐Akt, HIF‐2α, and VEGF expression. (F, G) A significant decrease in p‐Akt, HIF‐2α, and VEGF expression was seen in cells treated with single drug; however, the additive effect of drug combinations showed complete abolishment in HIF‐2α and VEGF as well as in phosphorylation of Akt at Ser^473^ expression compared with cells treated with single drug and control cells. GAPDH was used as a loading control. Further evidence of the effect of drug combinations on HIF‐2α function was measured by luciferase assay. (H) The additive effect of drug combinations caused a more than 90% decrease in the promoter activity of HIF‐2α compared with control cells using the Luciferase Reporter Assay System normalized to Renilla activity and measured by a luminometer. Data represent mean ± SEM (*n* = 4). Significant difference from control tissues is indicated by *P <* *0.01.

Since cell proliferation is necessary for tumor progression, we tested the effect of each drug or drug combinations on cell proliferation. 786‐O and ACHN cells were treated with serial concentrations of AICAR (0–10 mm), rapamycin (0–100 nm) or a combination of both drugs (2/20, 4/40, 10/100, mm/nm) for 72 h. Proliferation as assessed by [^3^H]‐thymidine incorporation assay demonstrated a dose‐dependent decrease for cells treated with each drug alone with a maximum of onefold decrease at the highest dose of each drug compared with non‐treated cells (Figs 2A,B and S2). In comparison, 2/20, 4/40, and 10/100, mm/nm AICAR/rapamycin combinations more potently inhibited cell proliferation, with the lowest dose of combined drugs suppressing proliferation eight‐ to ninefold (Figs 2C and S2). As expected, exposure to drug combinations (2 mm/20 nm, AICAR/rapamycin) also exhibited time‐dependence (Figs 2D and S2). Drug combinations also more effectively decreased cell proliferation proteins (PCNA and cyclin D1) compared with single‐drug treatments (Figs 2E and S3). These data indicate that combined rapamycin and AICAR treatments additively decrease cell proliferation and may be particularly efficacious in slowing tumor progression.

### 3.3 Drug combinations more effectively inhibit Akt activity and abolish HIF/VEGF

Hyperactivation of the major Pro‐survival signaling kinases Akt and mTOR is present in several types of tumors including RCC, and overexpression of HIF‐1α and HIF‐2α is linked to tumor progression of RCC. Therefore, we tested the effect of each drug or drug combinations on Akt phosphorylation at Ser^473^, and protein expression of HIF‐2α and VEGF in 786‐O and ACHN cells. Cells were treated with rapamycin (20 nm) or AICAR (2 mm) or drug combinations (20 nm rapamycin/2 mm AICAR) for 72 h. Cells treated with each drug alone showed a significant decrease in p‐Akt, HIF2‐α and VEGF protein expression (Figs 2F and S3). In contrast, combining the drugs abolished Akt phosphorylation (Figs 2F and S3), HIF‐2α, and VEGF protein expression (Fig. 2G), indicating that combination of the drugs has an additive effect to block activation of Akt/HIF‐2α/VEGF pathway.

### 3.4 Drug combinations are more effective in blocking Akt promoter activity

Akt promoter reporter plasmid was transfected and *Renilla* reporter plasmid as transfection control into HRCC and 786‐O cells as described in Materials and methods (Section 2). Cells were treated with rapamycin (20 nm), AICAR (2 mm) or drug combinations (20 nm rapamycin/2 mm AICAR) for 72 h. Figure 2H shows that although each drug treatment separately significantly decreased Akt promoter activity compared with control cells, drug combinations decreased Akt promoter activity by 95%, suggesting that the additive effect of the combined drugs is sufficient to almost completely block the promoter activity of a major Pro‐survival kinase involved in RCC.

### 3.5 Drug combinations block binding of HIF‐2α to Akt promoter

**Figure 3:**
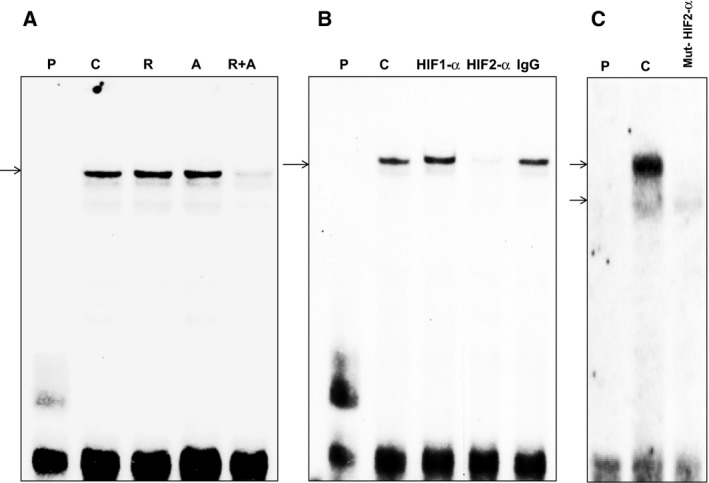
Combination of drugs reduces the binding of HIF‐2α to the Akt promoter element. (A) EMSA analysis of DNA probe corresponding to the putative HIF‐2α binding site in the Akt promoter was performed. Labeled probes were incubated with nuclear extracts isolated from 786‐O cells treated with rapamycin, AICAR or rapamycin+AICAR showed significant decrease binding of HIF‐2α to the Akt promoter. (B) Specificity of binding of HIF‐α to the Akt promoter was confirmed by adding HIF‐1α, HIF‐2α or IgG antibody to the reaction mixture. (C) Further confirmation of the binding site of HIF‐2 to Akt was obtained by incubation of the nuclear extract with mutated HIF‐2α probe. Data confirmed that HIF‐2α but not HIF‐1α was a part of the DNA/protein complex, and that the mutations in the binding sequence of HIF‐2 to Akt prevent the binding of HIF‐2 to the DNA/protein complex. These data provide new evidence that HIF2α is major transcription factor regulating cell survival kinase Akt.

To provide further evidence for the effectiveness of combining drugs on HIF‐2α function, we examined the effect of each drug and drug combinations on binding of HIF‐2α to the putative binding sequence in the Akt promoter using nuclear extracts from 786‐O cells. Nuclear extracts from control or cells treated with 2 mm AICAR, 20 nm rapamycin or 2 mm/20 nm AICAR/rapamycin were used for EMSA. Cells treated with each drug alone showed no significant changes, whereas cells treated with both drugs almost abolished the binding of HIF‐2α to the *Akt1* promoter (Fig. 3A). The specificity of HIF‐2α as a part of DNA/protein complex was tested by pre‐incubating the DNA/protein reaction with HIF1α or HIF‐2α or IgG antibody. Figure 3B shows that the DNA/protein complex was abolished when only HIF‐2α antibody was added in the reaction, which supports HIF‐2α as a major transcription factor that binds to the Akt promoter to activate cell survival (Fig. 3B). Mutation of the HIF‐2 primers was also used to confirm the binding site of HIF‐2α to the Akt promoter (see Materials and methods, Section 2). Mutations in HIF‐2α prevented the binding of HIF‐2 to the DNA/protein complex, confirming that HIF‐2α is essential to the formation of the complex (Fig. 3C). Taken together, these data support HIF‐2α as a major transcription factor that regulates the Pro‐survival kinase Akt.

### 3.6 Drug combinations additively decrease cell migration and invasion

**Figure 4:**
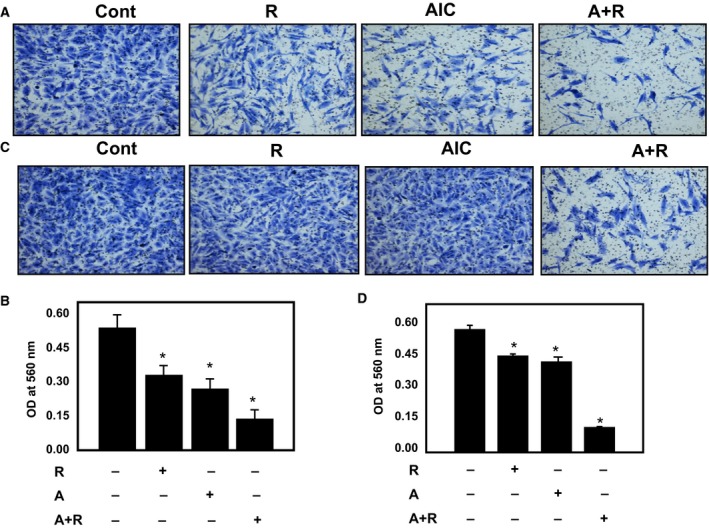
Drug combinations significantly decreased cell migration and cell invasion of kidney cancer cells. 786‐O cells treated with rapamycin (20 nm), AICAR (2 mm) or a drug combination (20 nm rapamycin/2 mm AICAR) for 72 h were added into the upper well chamber of a 96‐well plate. (A, B) Cells treated with drug combination have a significantly low number of migrated cells and (C, D) invaded cells compared with cells treated with a single drug or control cells. The total number of migrated and invaded cells was counted using counting software and the images of migrated or invaded cells were taken using a Nikon light inverted microscope. Data represent mean ± SEM (*n* = 4). Significant difference from control tissues is indicated by *P <* *0.01.

It is known that 786‐O cells have migration and invasion characteristics that promote metastasis. We next tested the effect of each drug or drug combination on cell invasion and cell migration. 786‐O cells treated with rapamycin (20 nm), AICAR (2 mm) or a drug combination (20 nm rapamycin/2 mm AICAR) for 72 h showed different patterns of cell migration and invasion. Figure 4A,B shows that cells treated with drug combinations have a significantly low number of migrated cells compared with cells treated with either drug alone. Kidney cancer cells with a metastatic ability can navigate through the extracellular matrix within a tissue and infiltrate neighboring tissues. Therefore, we tested the effects of individual drug treatment or drug combinations on cell invasion. Figure 4C,D shows that cells treated with drug combinations have a significantly lower number of invaded cells, indicating that this additive effect may prevent cell migration and invasion, and thus tumor metastasis.

### 3.7 Administration of single drug or a drug combination of rapamycin and AICAR to mice does not affect BW and is not nephrotoxic

**Figure 5:**
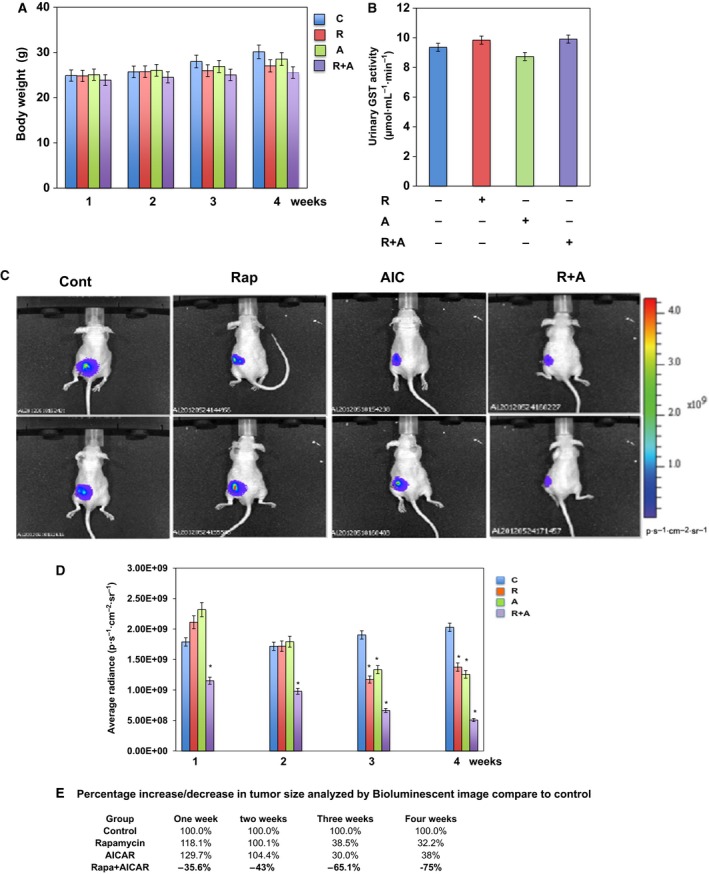
Drug combination and single‐drug treatment caused no change in BW and no toxicity in treated mice. (A) No changes in BW were detected between four groups of mice during 4 weeks of treatment with single or a drug combination. (B) Renal injury was determined by measuring the urinary excretion of GST in mice following treatment with rapamycin, AICAR or combination of rapamycin+AICAR for 4 weeks. GST activity is represented as μmol/mL/min. There were no significant changes between four groups of mice, indicating the absence of toxicity of treatment with a single drug or drug combinations. An additive effect of drug combinations resulted in significant decreases in kidney tumor size. (C) One million 786‐O cells expressing luciferase were injected in the kidney capsule of nude mice. Ten minutes prior to imaging, animals were injected with luciferin and the bioluminescent image signals were recorded using the Xenogen IVIS System. Representative images show the tumor in each of four groups. (D) Total photon radiance (p/s/cm^2^/sr) in the tumor area was measured by bioluminescent imaging and blotted to show the differences in photon flux/s between four groups. Photon data were calculated and the percentage of changes in tumor sizes between the four groups was summarized. (E) Data show that treatment with drug combinations resulted in a decreased tumor radiance of 75%, compared with 32.2% and 38% in mice treated with rapamycin and AICAR, respectively. Values represent the mean ± SEM (*n* = 5). Significant difference from control mice is indicated by *P <* *0.01.

To evaluate adverse effects due to drug toxicity, weights were recorded weekly while mice underwent drug treatment. Figure 5A displays the BW of mice before and during the 4 weeks of injection with rapamycin, AICAR, and rapamycin+AICAR. There was a slight increase in BW of all mice in all four groups, although no significant changes in BW were noted in mice just before and just after injection with each of the drugs individually or with a combination of the two (Fig. 5A). To evaluate for nephrotoxicity, urine was collected from all groups a day before sacrificing the mice to measure GST as an indicator of the loss of cell membrane integrity. There were no significant changes in the levels of urinary GST activity between mice treated with single drug or drug combinations compared with a control group of mice (Fig. 5B). These data provide preclinical evidence of the safety of combining rapamycin and AICAR as a novel treatment for kidney cancer.

### 3.8 Combining rapamycin and AICAR more effectively reduces kidney tumor size compared with individual drug treatments

We next sought to evaluate whether the additive effect of drug combinations observed in our *in vitro* experiments would have similar effects on reducing kidney tumor size *in vivo*. One million 786‐O cells stably expressing high luciferase activity under the Akt promoter were injected into one of the kidney capsules of 5‐week‐old nude mice. Figure 5C displays images of tumor size as indicated by the levels of emitted luminescence. Figure 5C demonstrates a significant decrease in the kidney tumor size of mice following 4 weeks of injection with drug combinations compared with mice injected with each drug alone or control mice. Summarized data of emitted luminescence from tumors measured as average radiance (p/s/cm^2^/sr) from each group of mice confirmed the increase in tumor size in control mice group vs. treated mice group (Fig. 5D). The emitted luminescence of the control mice group was considered 100% for all time points. We observed a significant increase in tumor size for the control mice group that was detected as early as 1 week after the injection of cancer cells and which continued to increase over the subsequent 4 weeks. In contrast, a significant decrease in emitted luminescence in the kidney tumors of mice treated with a combination of rapamycin and AICAR was detected after only 1 week of treatment compared with no changes in tumor size for mice treated with only one of the drugs at that same time point (Fig. 5D). A significant decrease in tumor radiance for mice treated with one drug (rapamycin, 38.5% and AICAR, 30%) and a drug combination (rapamycin+AICAR, 65%) was observed after 3 weeks of treatment compared with control mice (Fig. 5E), and a major additive effect of the drug combination of decreasing tumor size by 75% was present after 4 weeks of treatment; no significant decrease in tumor radiance was evident between 3 and 4 weeks for mice treated with only one of the drugs (Fig. 5E).

**Figure 6:**
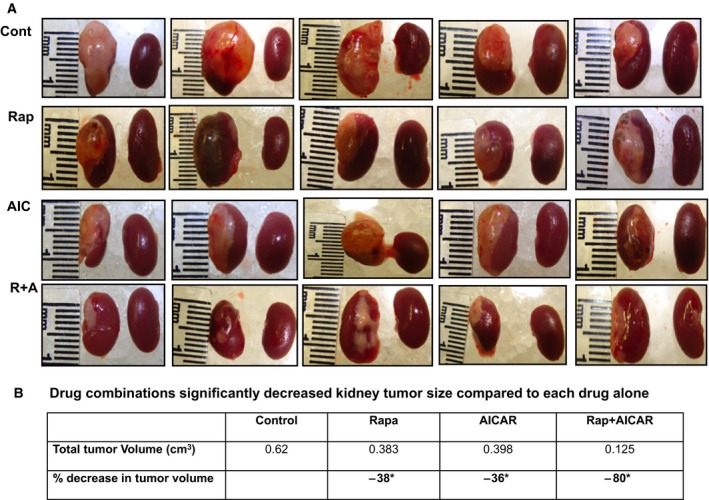
Drug combinations are more effective in reducing kidney tumorigenesis. A macroscopic view of all mice kidneys was examined and tumor size was measured along three axes as cm^3^ (length, width, and height). (A) Images of kidney from four groups of mice showing the tumor size in each group. (B) Data calculated from the tumor size from each group show that treatment with drug combination resulted in an 80% reduction in tumor size, treatment with rapamycin a 38% reduction, and treatment with AICAR a 36% reduction compared with control mice, further confirming the reduction of tumor size measured by bioluminescent imaging. Values represent the mean ± SEM (*n* = 5). Significant difference from control mice is indicated by *P <* *0.01.

To confirm the bioluminescence imaging data, mice were sacrificed after imaging, tumors were dissected, and gross and histological examinations were performed. Tumor size was measured along three axes (length, width, and height) and presented as cm^3^. Image and gross measurements of tumor size (Fig. 6A) were very close to the data obtained by bioluminescence imaging, with rapamycin decreasing tumor size by 38%, AICAR by 36%, and a drug combination by 80% compared with tumors from control mice (Fig. 6B). These findings corroborate our *in vitro* findings and demonstrate that combining rapamycin and AICAR has a potent additive effect on decreasing tumor progression in a preclinical mouse model of RCC.

### 3.9 Combining rapamycin and AICAR more effectively blocks cell proliferation and induces apoptosis in kidney tumors compared with individual drug treatments

**Figure 7:**
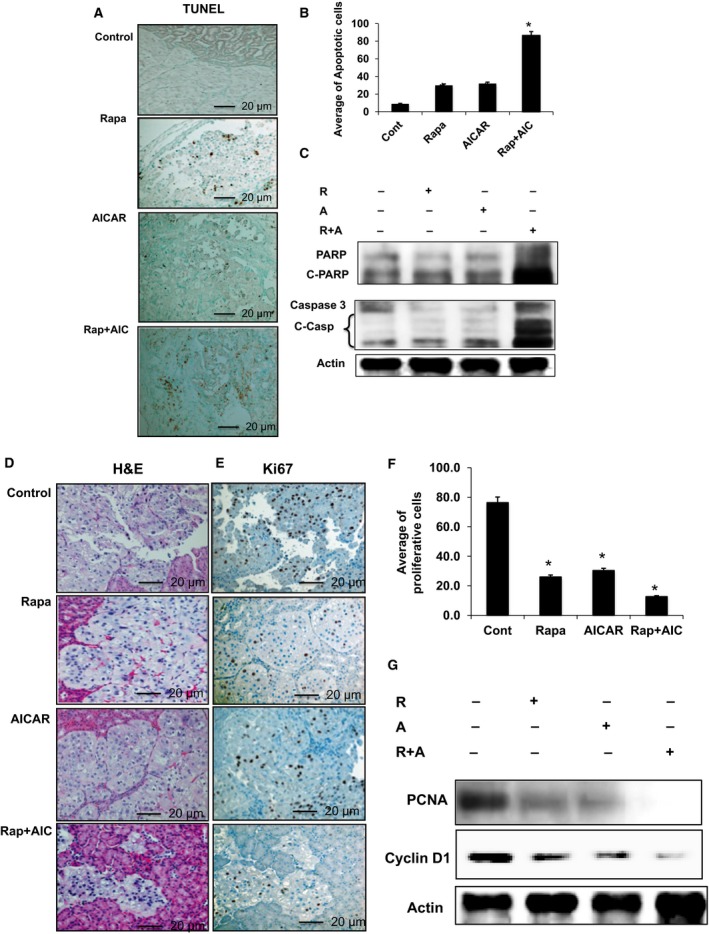
The additive effect of drug combinations strongly increased cell apoptosis, decreased cell proliferation, and abolished p‐Akt. (A) TUNEL assay was performed in kidney sections of four groups of mice and pictures taken with a light microscope. (A, B) Positive‐stained nucleus for TUNEL shows a significant increase in the number of positive cells in mice treated with drug combinations and less in groups treated with a single drug, compared with control mice. In addition, tumor kidney homogenates from all four mice groups were subjected to Western blot analysis. (C) Apoptosis proteins including cleavages of PARP and caspase 3 were measured and confirmed TUNEL data showing a significant increase of apoptosis in mice treated with drug combinations compared with mice treated with a single drug and control mice. (D) Hematoxylin and eosin staining in a tumor kidney section shows that ccRCC formed within the tumor tissue in all four groups. (E, F) Staining of the proliferative protein Ki67 shows a significant decrease in the number of positive‐stained nuclei in kidney tumors from mice treated with drug combinations compared with mice treated with either drug alone. (G) Western blot analysis performed in tumor homogenates from four groups of mice shows abolishment of PCNA expression, with a significant decrease in cyclin D1 in mice treated with drug combinations than in mice treated with a single drug and control mice. The scale bar size used for all images is 20 μm. Values represent the mean ± SEM (*n* = 5). Significant difference from control mice is indicated by *P <* *0.01.

TUNEL assays were performed on kidney tumor sections to evaluate the effects on apoptosis of treatment with rapamycin or AICAR vs. a combination of the two. The number of apoptotic cells in mice treated with the drug combination showed a larger number of apoptotic cells compared with tumors from mice treated individually with each drug or control mice (Fig. 7A,B). These data were confirmed in a Western analysis of kidney tumor homogenates from the four groups of mice with strong PARP, and caspase 3 cleavage was observed for tumors from mice treated with the drug combination compared with tumors from mice treated with each drug individually or control mice (Fig. 7C). Hematoxylin and eosin‐stained slides of the tumor demonstrated a clear cell (cc)RCC phenotype; reductions in tumors were histologically evident in treated mice compared with control mice (Fig. 7D). Data in Fig. 7E,F demonstrate a more prominent decrease in Ki67‐positive nuclei in tumors in mice treated with the drug combination than in mice treated with each drug alone; tumors from control exhibited the largest number of Ki67‐positive nuclei. A similar pattern was noted for the expression of proliferative protein markers (cyclin D1 and PCNA) by Western blot analysis. Figure 7G demonstrates that treatment with a combination of both drugs strongly decreased cyclin D1 and abolished PCNA expression compared with mice treated with each drug alone or control mice. These data suggest that the strong tumor regression observed in response to treatment with the drug combination is due to its additive effect on decreasing cell proliferation and increase cell apoptosis.

### 3.10 Combining rapamycin and AICAR potently inhibits Akt and decreases HIF‐2α/VEGF expression

**Figure 8:**
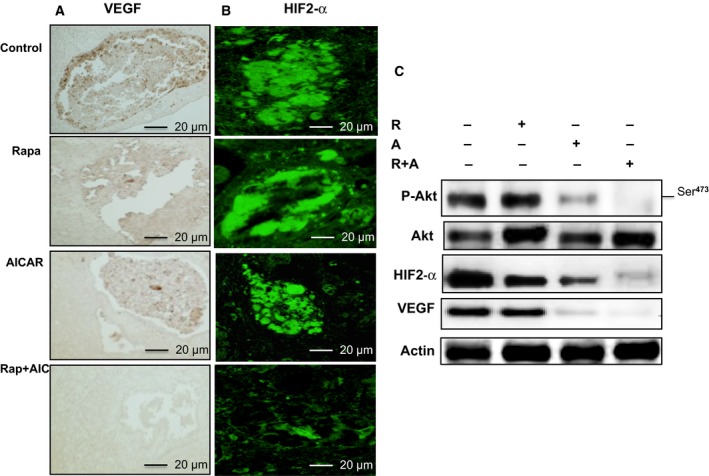
Additive effect of drug combinations strongly decreased HIF‐2α and VEGF expression. (A) Immunoperoxidase staining for VEGF and (B) immunofluorescence staining for HIF‐2α both show a significant decrease in protein staining in mice treated with drug combinations and mice treated with a single drug. (C) Western blot data show that drug combinations nearly abolished p‐Akt and significantly decreased expression of HIF‐2α and VEGF protein expression in tumor tissues compared with tumors from mice treated with a single drug and control mice. The scale bar size used for all images is 20 μm.

Because Akt is a major cell survival kinase in RCC, it is imperative to identify drugs that can target the kinase in order to inhibit cancer cell survival and control tumor progression. Hyperactivation of HIF‐2α is positively associated with the upregulation of vascular endothelial growth factor (VEGF), a key factor in tumorigenesis. Figure 8A,B demonstrates that tumors from mice treated with combined rapamycin and AICAR exhibited decreased staining for VEGF and HIF‐2α compared with tumors from mice treated with either drug individually or control mice. In addition, Western blot data in Fig. 8C confirmed that VEGF and HIF‐2α expression were significantly decreased in mice treated with both rapamycin and AICAR compared with mice treated with either drug alone or control mice. Taken together, these data indicate that combined treatment with rapamycin and AICAR in a mouse preclinical model of RCC additively reduces signaling through the Akt/HIF‐2α/VEGF pathway, which plays a key role in tumor progression and metastasis.

## 4 Discussion

No current clinical studies have used the combination of rapamycin and AICAR for the treatment of kidney cancer patients. Since rapamycin has been used for the treatment of several types of cancer, and AICAR has been approved for use in clinical studies, we investigated the combination of rapamycin+AICAR for the treatment of kidney cancer using established RCC cell lines (786‐O and ACHN) and tumor xenograft mouse model with the ccRCC cell line 786‐O cells. We found that the drug combination of rapamycin and AICAR had a strong additive effect in decreasing cell proliferation, increasing cell apoptosis, and abolishing Akt and HIF2α expression in RCC cell lines and primary ccRCC cells isolated from kidney cancer patient. We identified a putative HIF‐1/2α binding site in the *Akt1* promoter, and found that the drug combination abolished the binding of HIF‐2α to the *Akt1* promoter, which provides new evidence for HIF‐2α as a major transcription factor that is involved in activation of the cell survival kinase Akt. Importantly, the drug combination additively decreased cancer cell migration and invasion compared with treatment with either drug alone, which suggests that the drug combination may be useful for the prevention of RCC progression and metastasis.

Nephrotoxicity is a major adverse effect of anticancer drugs. The injection of rapamycin or AICAR or a combination of the two into mice for 4 weeks did not cause any significant changes in the levels of GST activity, a marker for renal dysfunction (Walshe *et al*., 2009), and there were also no changes in BW for any of the treatments, which is initial preclinical evidence for the safety of combination drug treatment for kidney cancer. We established a new 786‐O cell line expressing luciferase driven by the CMV promoter that formed large tumors that could be followed by bioluminescence imaging following injection into the kidney capsules of nude mice. Treatment with the drug combination for 4 weeks significantly decreased tumor sizes in mice as early as 1 week (35.6%) compared with control mice, and sharply decreased their sizes after 3 weeks (to −65.1%) and 4 weeks (to −75%). The gross tumor size of the mice in all four groups was measured after they were sacrificed and confirmed the additive effect of the drug combination in decreasing kidney tumor progression compared with single‐drug treatment. Drug combinations of rapamycin and AICAR increased tumor cell apoptosis, decreased cell proliferation, and sharply decreased Akt phosphorylation and HIF‐2α in ccRCC as well as VEGF in tumors compared with single‐drug treatment. These preclinical findings support the potential of drug combination therapy with rapamycin and AICAR to additively reduce kidney tumor progression.

mTOR, a serine/threonine kinase belonging to the phosphatidylinositol kinase‐related kinase family, plays a central role in regulating cell growth, proliferation, and survival, in part by regulating translation initiation (Dowling *et al*., 2010; Inoki *et al*., 2005). mTOR signaling pathways are constitutively activated in many types of human cancer (Wagle *et al*., 2014a,b; Thoreen *et al*., 2009), and targeting the kinase has emerged as an important approach in cancer therapy. Early clinical trials have shown that VHL‐related kidney tumors regress in response to treatment with rapamycin, a direct target of inhibition of mTORC1 (Lane and Breuleux, 2009). Our findings showed that blocking mTORC1 with rapamycin increased cell apoptosis, decreased cell proliferation, inhibited the Akt/HIF2‐α/VEGF pathway, and impaired the migration and invasion of 786‐O cells. Moreover, our *in vivo* studies showed that rapamycin treatment reduced the tumor size of xenografted mice by 32.2% compared with control mice. On the other hand, mTORC2 promotes the activation of the cell survival kinase Akt via its phosphorylation of Ser^473^ in response to growth factors (Inoki *et al*., 2005). Activation of AMPK by AICAR also increased apoptosis, decreased cell proliferation, inhibited the Akt/HIF2‐α/VEGF pathway in 786‐O cells, and reduced the tumor size in xenografted mice by 38% compared with control mice.

As a sensor of nutrients, AMPK is an important upstream signaling mediator in the regulation of the mTOR pathway (Agarwal *et al*., 2015). Activation of AMPK using metformin or AICAR markedly inhibited the invasion of UOK262 cells, a cell line derived from a patient with recurrent hereditary leiomyomatosis and renal cell cancer kidney cancer, by modulating HIF‐1α, which raises the potential for targeted therapeutic approaches for the treatment of renal kidney cancer‐associated kidney cancer (Tong *et al*., 2011). We found that AICAR reduced HIF‐2α expression in 786‐O cells as well as their ability to invade. We previously demonstrated that activation of AMPK by AICAR results in decreased p70S6K phosphorylation and increased expression of the DNA repair protein OGG1, and that rapamycin induced AMPK activation, which leads to increased OGG1 expression in 786‐O cells (Habib *et al*., 2010a,b). Our *in vivo* and *in vitro* findings showed that blocking mTOR and activating AMPK prevented tumor progression in a tumor xenograft mouse model. In agreement with our findings, it was recently reported that a new mTOR kinase inhibitor, WYE‐687, increased apoptosis and blocked activation of both mTORC1 and mTORC2 through the feedback activation of p‐Akt in RCC cells, and oral administration of WYE‐687 potently suppressed tumor growth in nude mice injected with 786‐O cells (Pan *et al*., 2017). Induction of VEGF expression is dependent on HIF‐mTOR activation, which indicates a critical function of the HIF‐mTOR pathway in regulating angiogenesis (Land and Tee, 2007). We found that rapamycin or AICAR reduced VEGF protein expression, but combining both resulted in near abolishment of VEGF expression in VHL‐deficient human cells. Increased expression of VEGF is associated with malignant progression and a poor treatment outcome (Caoy *et al*., 2012). These findings suggest that suppressing the HIF‐mediated, hypoxia‐induced VEGF gene pathway may be an important therapeutic strategy for RCC. Indeed, targeting the VEGF/mTOR pathway with a combination of mTOR (temsirolimus) and an antibody directed against VEGF (bevacizumab) is being evaluated in a Phase II clinical trial for patients with RCC (Gordan *et al*., 2008). A significant number of *VHL* ^−/−^ RCC express only HIF‐2‐α resistant to mTORC1 inhibitors due to their inability to suppress HIF‐2α expression (Battelli and Cho, 2011). Our finding that a drug combination of rapamycin and AICAR resulted in an 80% decrease in tumor size compared with 38% with rapamycin or 36% with AICAR, underscores the potential utility of this drug combination for kidney cancer, including VHL^−/−^ RCCs.

## 5 Conclusion

Our data show no significant effect of single and combined drug treatments with rapamycin and AICAR on BW and no toxicity to the kidneys of nude mice. We found that combining the two drugs had a strong additive effect in inhibiting the proliferation of RCC cells and increasing apoptosis; the drug combination blocked the activity of the Pro‐survival kinase Akt and inhibited tumor progression. Our results suggest that tumor progression is decreased by inhibition of the Akt/HIF‐2α/VEGF/mTOR pathway. These results provide preclinical evidence for the safety of combining rapamycin and AICAR to repress RCC tumor progression, and demonstrate for the first time in a preclinical mouse model of RCC that this drug combination additively reduces kidney tumor progression. Finally, since rapamycin and AICAR are already being used in patients, these findings suggest that evaluation of this drug combination in patients with kidney cancer may be warranted.

## Author contributions

Conception and design: SLH, BL, and SL. Development of methodology: SL, BL, and SLH. Acquisition of data (providing animals, acquiring and managing patients, providing facilities, etc.): SLH and SL. Analysis and interpretation of data (e.g. statistical analysis, biostatistics, computational analysis): SLH, SL, and EAM. Writing, review, and/or revision of the manuscript: SLH, SL, and EAM. Administrative, technical or material support (i.e. reporting or organizing data, constructing databases): SLH and SL. Study supervision: SLH and SL.

## Supporting information


**Fig. S1.** Significant increase in number of apoptotic cells is drug concentration and time exposure dependent in ACHN cells.
**Fig. S2.** Drug combinations significantly decreased cell proliferation is depending on the drug concentration and time of exposure in ACHN cells.
**Fig. S3.** A combination of drugs significantly increased PARP cleavage, decreased proliferative proteins and abolished Akt phosphorylation.
