# Supplementary material for: Preclinical evidence of the enhanced effectiveness of combined rapamycin and AICAR in reducing kidney cancer
Source: Mol Oncol. 2018 Oct 12;12(11):1917–34. doi: 10.1002/1878-0261.12370 (PMC6210038; doi:10.1002/1878-0261.12370)
Supplement: Supplementary file 1 — Fig. S1. Significant increase in number of apoptotic cells is drug concentration and time exposure dependent in ACHN cells. Fig. S2. Drug combinations significantly decreased cell proliferation is depending on the drug concentration and time of exposure in ACHN cells. Fig. S3. A combination of drugs significantly increased PARP cleavage, decreased proliferative proteins and abolished Akt phosphorylation. [file MOL2-12-1917-s001.ppt]

## Slide 1
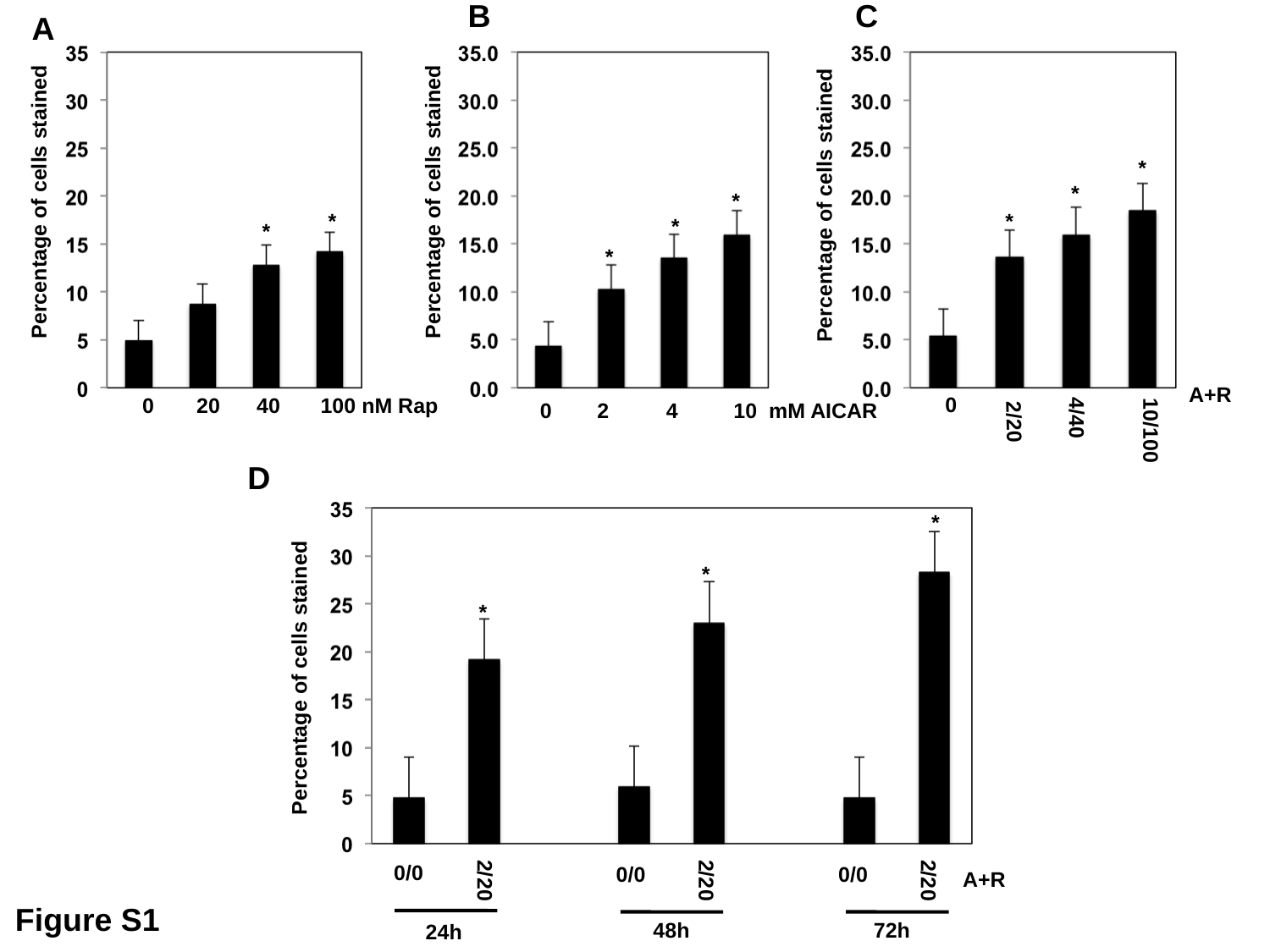

B
C
A
*
*
*
Percentage of cells stained
Percentage of cells stained
Percentage of cells stained
*
*
*
*
*
A+R
 0
 0
 20
 40
 100 nM Rap
 0
 2
 4
 10 mM AICAR
4/40
2/20
10/100
D
*
 *
*
Percentage of cells stained
0/0
0/0
0/0
A+R
2/20
2/20
2/20
Figure S1
48h
72h
24h

## Slide 2
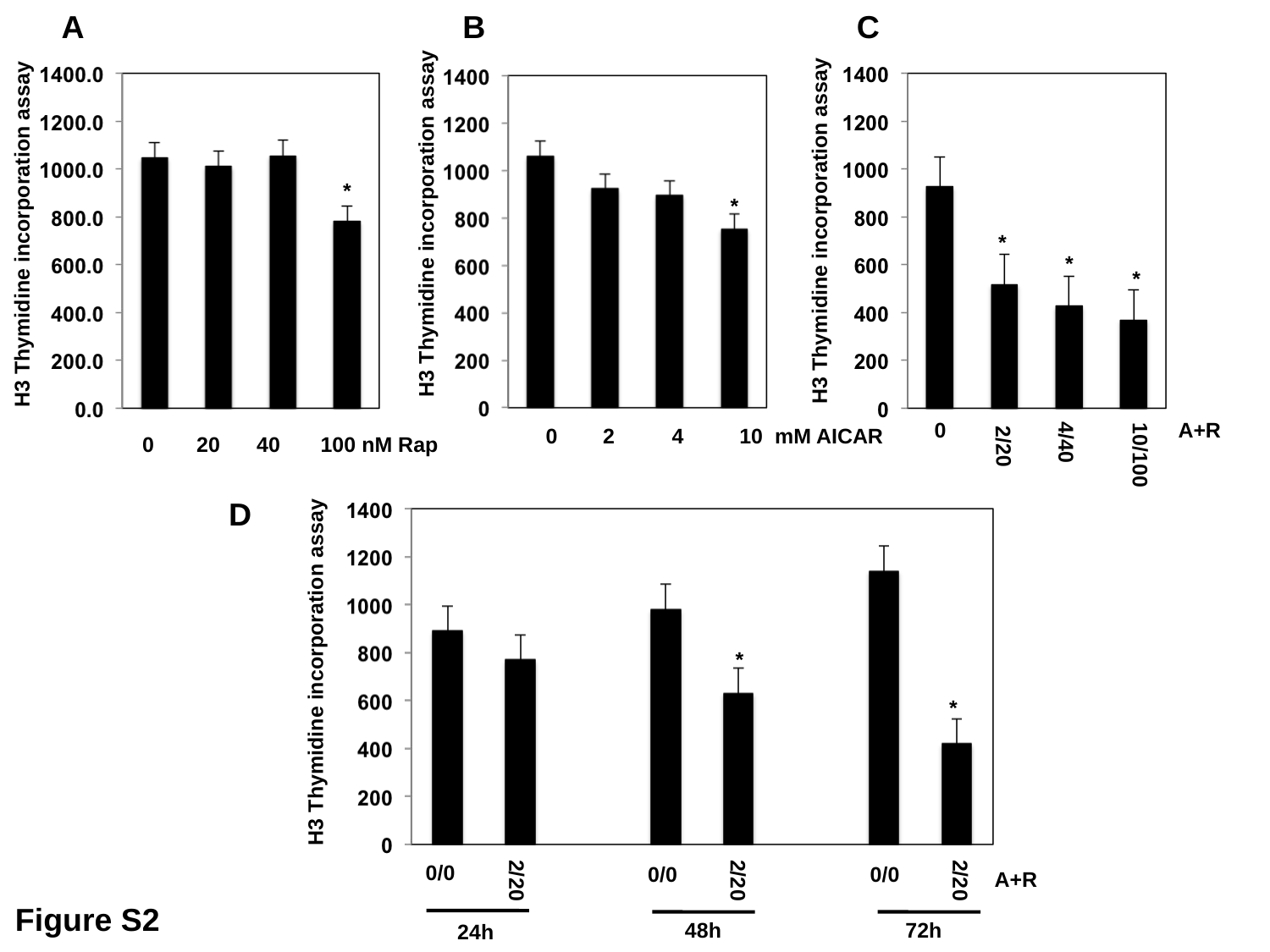

A
B
C
*
*
H3 Thymidine incorporation assay
H3 Thymidine incorporation assay
H3 Thymidine incorporation assay
*
*
*
 0
A+R
 0
 2
 4
 10 mM AICAR
4/40
2/20
 0
 20
 40
 100 nM Rap
10/100
D
*
H3 Thymidine incorporation assay
*
0/0
0/0
0/0
A+R
2/20
2/20
2/20
Figure S2
48h
72h
24h

## Slide 3
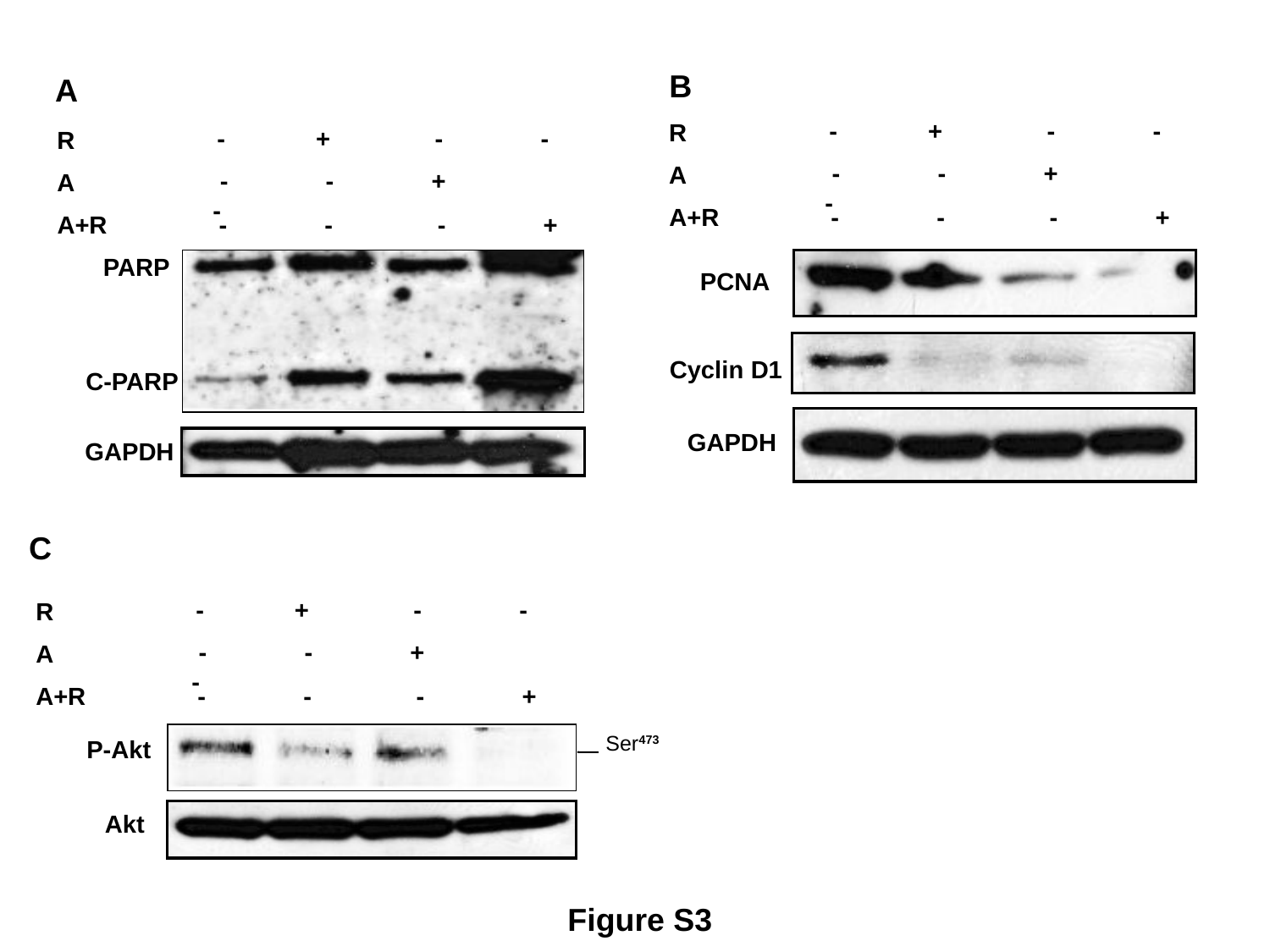

B
A
- + - -
 - - + -
- - - +
R
A
A+R
- + - -
 - - + -
- - - +
R
A
A+R
 PARP
PCNA
Cyclin D1
 C-PARP
GAPDH
 GAPDH
C
- + - -
 - - + -
- - - +
R
A
A+R
Ser473
P-Akt
Akt
Figure S3
