# Supplementary material for: Preclinical evidence of the enhanced effectiveness of combined rapamycin and AICAR in reducing kidney cancer
Source: Mol Oncol. 2018 Oct 12;12(11):1917–34. doi: 10.1002/1878-0261.12370 (PMC6210038; doi:10.1002/1878-0261.12370)
Supplement: Supplementary file 2 [file MOL2-12-1917-s002.docx]

**Supplementary data:**

**Figure legends:**

**Figure S1. Significant increase in number of apoptotic cells is drug concentration and time exposure dependent in ACHN cells.** Serial concentrations of (A) rapamycin (0-100nM), (B) AICAR (0-10mM) (C) drug combinations (0/0-2/20,4/40 and 10/100 mM/nM) show that increased in number of apoptotic cells is dose-dependent using annexin V-FITC conjugated to PI by flow cytometry. (D) Treatment of the cells with drug combinations for 24, 48, and 72hrs show that increased in number of apoptotic cells is time dependent. Data represent means±SE (n=4). Significant difference from control tissues is indicated by * *P* < 0.01.

**Figure S2. Drug combinations significantly decreased cell proliferation is depending on the drug concentration and time of exposure in ACHN cells.** Serial concentrations of (A) rapamycin (0-100nM), (B) AICAR (0-10mM) (C) drug combinations (0/0-2/20,4/40 and 10/100 mM/nM) show that decreased in cell proliferation is dose-dependent using ^3^H-thymidine incorporation assay. (D) Treatment of the cells with drug combinations for 24, 48, and 72hrs show that significant decreased in cell proliferation is time dependent. Data represent means±SE (n=4). Significant difference from control tissues is indicated by * *P* < 0.01.

**Figure S3**: **A combination of drugs significantly increased PARP cleavage, decreased proliferative proteins and abolished Akt phosphorylation.** (A) Apoptotic data was confirmed in cells by measuring apoptotic protein expression. Lysates from cells treated with rapmycin (20nM), AICAR (2mM), rapamycin+AICAR (20nM/2mM) for 72hrs were subjected to Western blot analysis. (A) Significant increase was detected in cleavage of PARP at 85 KDa in ACHN cells treated with drug combinations compared to cells treated with each drug alone for 72hrs. (B) Cell proliferation data was confirmed in cells by measuring proliferative proteins. Significant decrease in expression of PCNA and cyclin D1 was detected in cells treated with single drug while abolishment of expression of both proteins detected in cells treated with drug combinations providing evidence of the additive effect of drug combinations on reducing cell proliferation. (C) **A combination of drugs abolished Akt survival kinase**. Significant decreased in p-Akt expression in cells treated with single drug while additive effect of drug combinations showed complete abolishment of phosphorylation of Akt at Ser^473^ expression compared to cells treated with single drug and control cells. GADPH was used as a loading control.
